# Challenges in diagnosing head and neck cancer in primary health care

**DOI:** 10.1080/07853890.2020.1802060

**Published:** 2020-08-05

**Authors:** Markus Nieminen, Katri Aro, Antti Mäkitie, Vappu Harlin, Satu Kainulainen, Lauri Jouhi, Timo Atula

**Affiliations:** a Department of Otorhinolaryngology – Head and Neck Surgery, University of Helsinki and Helsinki University Hospital, Helsinki, Finland; b Department of Clinical Sciences, Intervention and Technology, Division of Ear, Nose and Throat Diseases, Karolinska Institutet, Karolinska University Hospital, Stockholm, Sweden; c Research Program in Systems Oncology, Faculty of Medicine, University of Helsinki, Helsinki, Finland; d Department of Social Services and Health Care, City of Helsinki, Helsinki, Finland; e Department of Oral and Maxillofacial Surgery, University of Helsinki and Helsinki University Hospital, Helsinki, Finland

**Keywords:** Head and neck neoplasm, primary health care, incidence, epidemiology, time-to-treatment, treatment delay

## Abstract

**Background:**

Early diagnosis of head and neck cancer (HNC) will improve patient outcomes. The low incidence of HNC renders its detection challenging for a general practitioner (GP) in primary health care (PHC).

**Patients and methods:**

To examine these challenges, our cohort consisted of all patients visiting PHC centres in the City of Helsinki in 2016. We chose 57 ICD-10 codes representing a sign or symptom resulting from a possible HNC and compared data for all new HNC patients.

**Results:**

A total of 242,211 patients (499,542 appointments) visited PHC centres, 11,896 (5%) of whom presented with a sign or symptom possibly caused by HNC. Altogether, 111 new HNCs were diagnosed within the Helsinki area, of which 40 (36%) were referred from PHC. The median delay from the initial PHC visit to the referral to specialist care was 5 days, whereby 88% of patients were referred within one month.

**Conclusions:**

Despite the low incidence of HNC and the large number of patients presenting with HNC-related symptoms, GPs working in PHC sort out potential HNC patients from the general patient group in most cases remarkably effectively.

**KEY MESSAGES**
- For every head and neck cancer (HNC) patient encountered in the primary health care, a general practitioner (GP) will meet approximately 6000 other patients, 100 of whom exhibit a sign or a symptom potentially caused by a HNC.
- Despite the low incidence of HNC, GPs referred patients to specialist care effectively, limiting the median delay from the initial appointment to referral to only 5 days.

## Introduction

Any delay in the diagnosis and treatment of head and neck cancer (HNC) might affect patient outcome [1–5]. The worldwide incidence of HNC was 11.6/100,000 in 2018 [6]. Given this low incidence, the proportion of HNC patients compared to the patient population a single GP encounters while working in a public primary health care (PHC) center may remain quite small. Furthermore, HNC might cause a variety of symptoms that commonly accompany benign conditions as well, such as pain in the throat. In a recent Swedish study of 114,538 randomly selected adults from the general population, the prevalence of voice problems (tiring, strain or hoarseness) was 16.9% [7]. Another population-based study among randomly selected adults from the United States found that symptoms indicative of chronic rhinosinusitis were reported by 11.5% of participants, 94–97% of whom experienced nasal congestion and/or obstruction [8]. These factors represent an immense challenge to GPs when sorting potential cancer patients from the general patient population.

The European Head and Neck Society launched the Make Sense Campaign (MSC) in 2013 in order to inform the general public and PHC personnel about HNC [9]. The MSC campaign website provides information about risk factors and symptoms associated with HNC [9], while introducing a “1-for-3” rule regarding symptoms and their persistence. Symptoms include: 1. a sore tongue, non-healing mouth ulcers and/or red or white patches in the mouth; 2. pain in the throat; 3. persistent hoarseness; 4. painful and/or difficulty swallowing; 5. a lump in the neck; and 6.a blocked nose on one side and/or bloody discharge from the nose. The 1-for-3 rule states that, if a patient experiences one or more of the six most common HNC-related symptoms for more than three weeks, the GP should refer the patient to a head and neck specialist.

The background population of patients who contact a GP with a symptom potentially caused by HNC and among whom a GP needs to identify suspected HNC patients for further referral remains largely unexamined. Here, we aimed to study this background population in order to investigate the challenges encountered by a GP related to detecting HNC patients. We hypothesised that due to the rarity of HNC, their diagnostics in PHC is difficult. We also aimed to provide a rough estimate for the likelihood that a PHC patient of a certain age with a specific symptom has HNC.

## Patients and methods

We retrospectively investigated the total number of patients attending outpatient visits at all 28 PHC centres in the City of Helsinki, with a population of 635,181 in 2016, excluding emergencies. Data were collected from the centralised electronic database managed by the Social Services and Health Care Division of Helsinki, which records every visit to a nurse or a GP. The database contains all patient information recorded through the patient record system Pegasos, that was used in all 28 PHC centres. From this database, it is possible to retrieve anonymous data from patient appointments that include the patients’ age, sex and the ICD-10 diagnosis code used. In Finland, it is compulsory for a GP to select at least one diagnosis using the ICD-10 codes most accurately describing the reason for each PHC visit. However, it is not possible to automatically gather any information written in text form.

We selected 57 ICD-10 codes we identified as representing a sign or symptom potentially caused by HNC, examining how often they were used in our patient population (Table 1). Data were then grouped according to patient sex and age. We were unable to gather information on the duration of symptoms. We chose 2016 as the year for our analysis, since we had previously meticulously examined different delays experienced by our new HNC patients during that year, including patient-related delays and PHC delays [10].

**Table 1.** Total number of patients that visited a primary health care centre with a head and neck cancer-related symptom.

Sign or symptom		ICD-10 codes used	Total number of patients (M/F^b^%)	Number of patients, M/F			Number of HNC patients, M/F (*n* = 33^d^)		
				Age			Age		
Symptoms related to HNC^a^				<40	40–59	>60	<40	40–59 (%^c^)	>60 (%^c^)
1.	Sore tongue, non-healing mouth ulcers and/or red or white patches in the mouth	K12, K12.01, K12.10, K12.18, K13.2-13.4, K13.6, K13.7, K13.78, K13.79, K14, K14.0-09, K14.6, K14.60-69, K14.88, K14.9	306 (37.6/62.4)	50/60	31/31	34/100	0/0	1/1 (3.2/3.2)	1/2 (2.9/2.0)
2.	Pain in the throat	R07.0	1157 (35.1/64.9)	257/451	79/149	70/151	0/0	1/0 (1.3/0.0)	2/1 (2.9/0.7)
3.	Hoarseness	J04, J04.0, R49.0, R49.8	976 (36.6/63.4)	231/316	38/143	88/160	0/0	0/0 (0.0/0.0)	2/0 (2.3/0.0)
4.	Painful and/or difficulty swallowing	J02, J02.9, J03, J03.9, J31.2, R13	3004 (40.4/59.6)	937/1318	137/242	140/230	0/0	1/0 (0.7/0.0)	3/0 (2.1/0.0)
5.	Lump in the neck	D23, D23.4, I88, I88.8, I88.9, R22, R22.0, R22.1, R59.0, R59.9	2267 (34.8/65.2)	387/600	168/355	233/524	0/0	1/0 (0.4/0.0)	7/2 (3.0/0.4)
6.	Blocked nose and/or bloody discharge from the nose	J31.0, J33, J33.0, J33.9, R04.0	1172 (47.9/52.1)	260/227	99/119	202/265	0/0	1/1 (1.0/0.8)	1/1 (0.5/0.4)
7.	Difficulty breathing and/or haemoptysis	R06, R06.0, R06.1, R04.2, R58	3014 (38.3/61.8)	305/541	221/262	628/1057	0/0	1/0 (0.5/0.0)	2/1 (0.3/0.1)
	Total		11,896 (38.6/61.4)	2427/3513	773/1301	1395/2487	0/0	6/2 (0.8/0.2)	18/7 (1.3/0.3)

^a^Head and neck cancer (symptoms 1–6 are part of the Make Sense Campaign).

^b^Male/female.

^c^The percentage of cancer in this age and sex group.

^d^No ICD-10 diagnosis code or the code recorded was not relevant to the case for seven patients.

In Helsinki, all HNC patients are referred to and treated at our university hospital (either in the Department of Otorhinolaryngology – Head and Neck Surgery or in the Department of Oral and Maxillofacial Surgery). The treatment modality for all HNC patients in the Helsinki and the surrounding region (Uusimaa), is assessed in multidisciplinary tumour board meetings, which are held weekly. We investigated all 111 new HNC patients diagnosed from the population of Helsinki in 2016. This area covers about one-third of the total referral population of our university hospital. We manually collected data on patients’ health-seeking behaviour, the number of visits to a doctor before the referral, the place from which the referral was made to specialist care (SC), the delay from the initial PHC visit until the referral to SC and the ICD-10 code for the appointment from hospital records. Additionally, regarding patients who were referred from PHC (*n* = 40), we collected their data from the patient record system that was used in the PHC centres (Pegasos). Patient-related variables included age and sex.

The Research Ethics Board at the Hospital District of Helsinki and Uusimaa approved the study design (record number: 398/13/03/02/15) and an institutional permit was granted. The Helsinki Social and Health Care Services also approved the study protocol.

## Results

### Outpatient visits to primary health care

A total of 242,211 patients visited a GP in PHC in the City of Helsinki in 2016, resulting in a total of 499,542 outpatient visits. Among these patients, 55.0% were over 40  years old and 60.2% were female. In December 2016, 380 GPs were concurrently working at PHC centres in the City of Helsinki.

**Figure 1.**
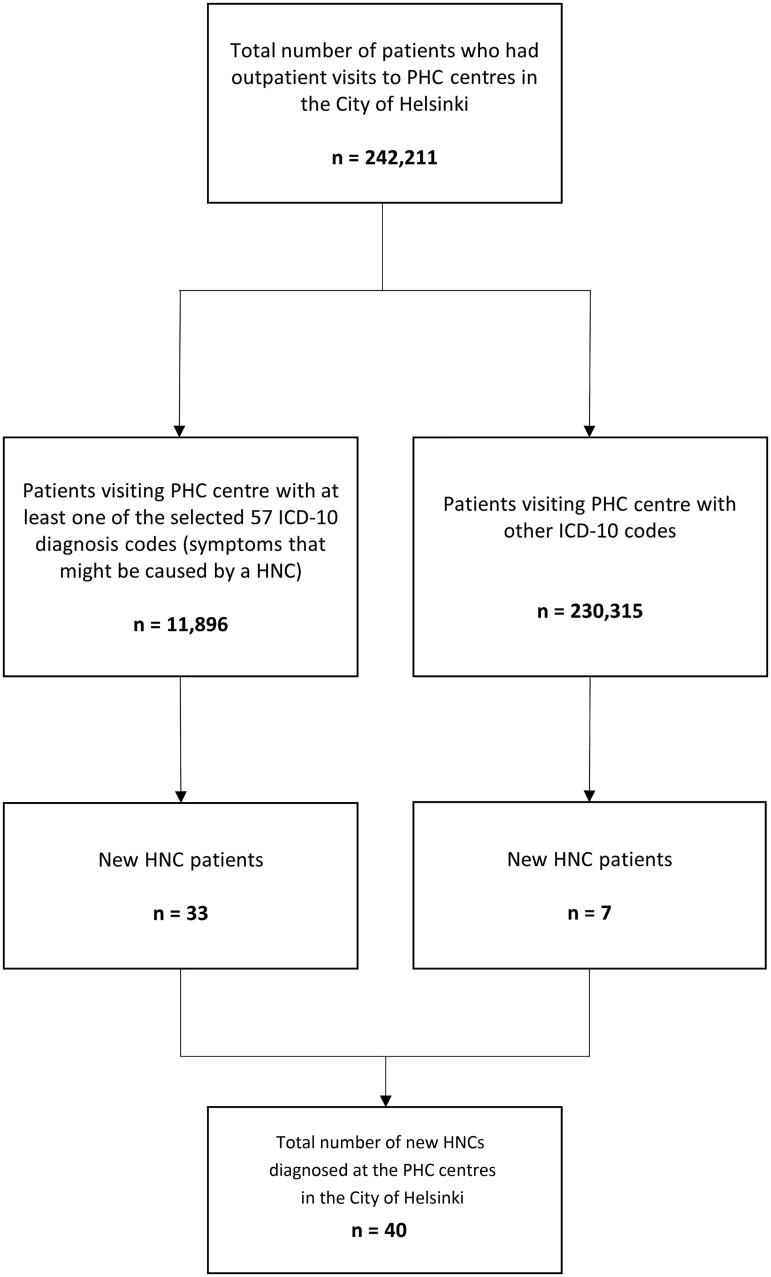
The total number of patients that visited PHC centres in the City of Helsinki in 2016 out of which 40 new HNCs were diagnosed at the PHC centres.

The total number of patients who visited a GP and had a symptom potentially caused by HNC reached 11,896 (Figure 1). Among these patients, 61.4% were female, 38.6% were male and 50.1% were over 40 years old. Table 1 summarises the distribution of symptoms, ICD-10 codes and the corresponding number of patients for each category. The symptoms mentioned in MSC do not include difficulties breathing and/or haemoptysis. The total number of patients who visited a GP presenting with a symptom mentioned in the MSC reached 8882 (61.3% female, 38.7% male), of whom 42.6% were over 40 years old.

### Head and neck cancer patients in the city of Helsinki

**Figure 2.**
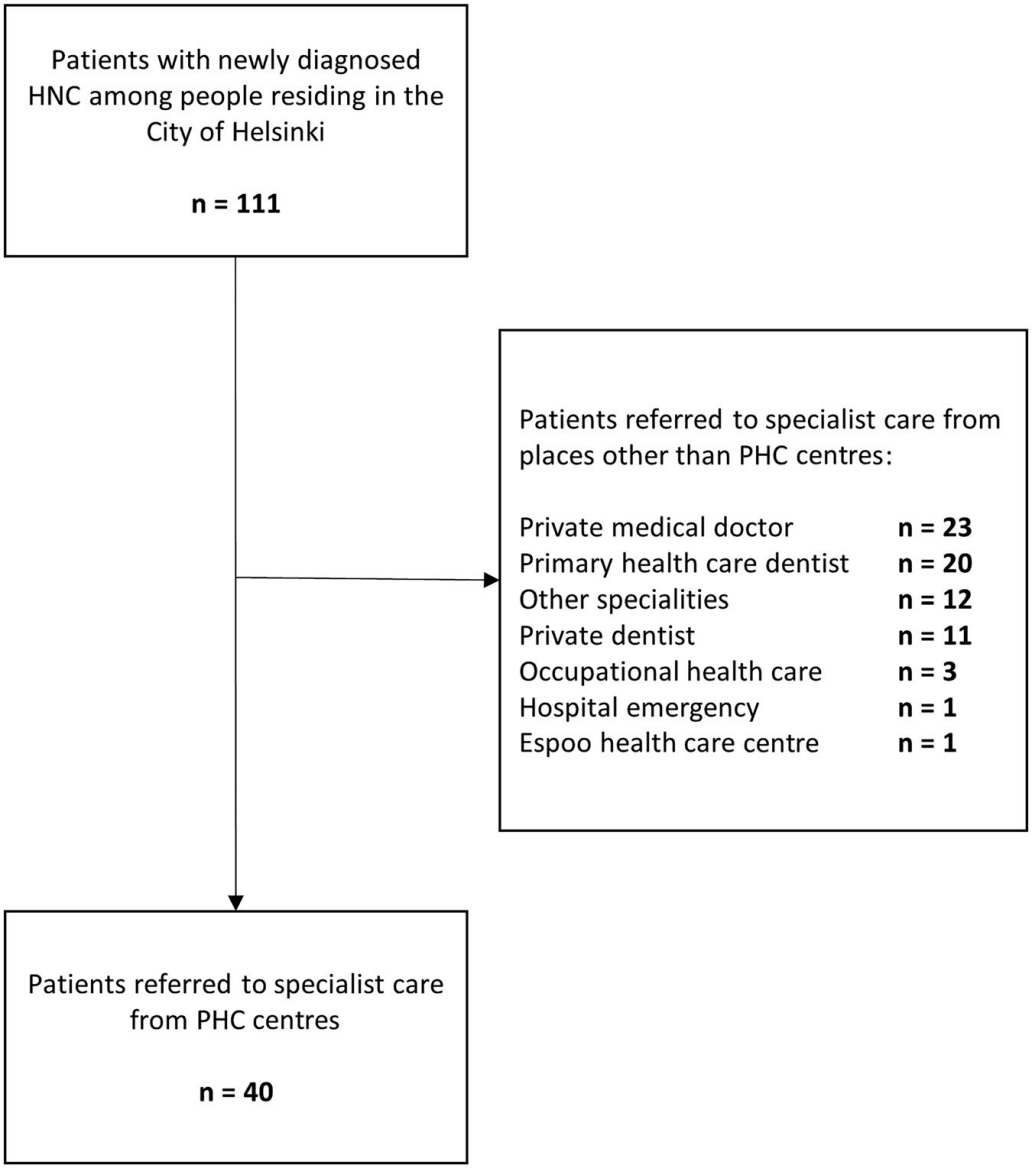
Places of referral of all new HNC cancer patients (*n* = 111) in the City of Helsinki in 2016.

In 2016, altogether 111 new HNCs were diagnosed among residents of the City of Helsinki. Public PHC centres referred 40 (36.0%) of them to SC (Figure 2). The remaining patients were referred from the private sector (*n* = 23; 20.7%), a public dentist (*n* = 20; 18.0%), other hospital specialties (*n* = 12; 10.8%), a private dentist (*n* = 11; 9.9%), occupational health care (*n* = 3; 2.7%) or a hospital emergency unit (*n* = 1; 0.9%). One patient visited a public PHC centre in a neighbouring city.

A total of 40 new HNC patients were referred from a public PHC centre to SC. Among these patients, 27 (67.5%) were male and 13 (32.5%) were female with a mean age of 64 (range, 45–80 years). The most common cancer sites among these patients were the oropharynx (*n* = 15; 37.5%), oral cavity (*n* = 7; 17.5%), hypopharynx (*n* = 6; 15%), nose and nasal cavity (*n* = 5; 12.5%), larynx (*n* = 4; 10%), unknown primary (*n* = 2; 5%) and major salivary glands (*n* = 1; 2.5%). All cancer sites according to the place of referral appear in Table 2.

**Table 2.** Number of HNC patients diagnosed in the Helsinki area in 2016 according to site and place of referral.

			Referral		
Tumour site	Total number	PHC^a^ centre	Private MD^b^	Dentist	Other
Oral cavity	45	7	3	29	6
Oropharynx	31	15	10	2	4
Larynx	10	4	3	0	3
Hypopharynx	9	6	2	0	1
Nose and nasal cavities	8	5	2	0	1
Major salivary glands	5	1	3	0	1
Unknown primary	3	2	0	0	1
Total	111	40	23	31	17

^a^Primary health care.

^b^Medical doctor.

The most common symptom among the 40 patients referred from PHC (according to the ICD-10 diagnosis code used at the visit) was a lump in the head and neck site (*n* = 10; 25%). Other symptoms listed in order of frequency included: non-healing mouth ulcers and/or red or white patches in the mouth (*n* = 5; 12.5%); pain in the throat (*n* = 4; 10%); painful and/or difficulty swallowing (*n* = 4; 10%); blocked nose and/or bloody discharge from the nose (*n* = 4; 10%); difficulties breathing and/or haemoptysis (*n* = 4; 10%); and hoarseness (*n* = 2; 5%). Three patients (7.5%) did not have any diagnosis code registered at their initial visit to PHC and 4 (10%) patients had an ICD-10 diagnosis code that was not relevant or was too general compared to their symptoms.

Half (*n* = 20; 50%) of the patients were referred to SC during the initial visit to the PHC centre, 17 patients at the second visit and 3 patients during the third visit. Two thirds of these additional visits (65%) were related to an ultrasound and fine-needle sample, while the remaining were clinical follow-up or other visits. Only one patient who initially visited a public PHC centre subsequently sought medical advice from the private sector.

The median doctor-related PHC delay (from the initial visit at PHC to the referral to SC) was 5 days (mean, 19 days; range, 0–248 days). More than half (57.5%) of the patients were referred to SC within a week, 72.5% within 2 weeks and 87.5% within 1 month. Only three patients had a PHC delay of more than 2 months.

## Discussion

In the present patient population consisting of patients residing in the Helsinki area, a new HNC was detected roughly once in every 12,500 visits or in every 6000 patients seen in PHC. Yet, the median doctor-related PHC delay was no more than 5 days, and half of the patients were referred to SC during the initial visit. The number of patients who presented with a similar symptom potentially caused by HNC remains largely unexamined. In our study, we collected data from all patients who visited a PHC centre in the City of Helsinki in 2016, amounting to a total of 242,211 patients. Among these patients, we identified 40 new HNC cases. To our knowledge, this is the only study that has thoroughly investigated the background population of patients among whom a GP needed to identify potential HNC patients for further referral.

In Finland, the management of all HNC patients is regulated by governmental authorities, organized by the public health care system and centralized at five university hospitals. Patients can either seek medical care through public PHC centres or private sector. Additionally, all employers must provide occupational health care services to their employees which are organized either through public or private sector. Public health care is almost entirely funded through tax collection by the municipality that the patient belongs to. Total payment limit for any health service is 683 euros during a calendar year, after which all care is entirely free for the patient. Private health insurances are not widely used because of good availability and low cost of public PHC and occupational health care. All sectors can similarly refer patients to public SC unit for further investigation and treatment of potential HNC.

In Finland, all head and neck malignancies are discussed at weekly multidisciplinary tumour board meetings held at the five university hospitals. Private treatment in the field of HNC does not exist; even the most minor malignancies are referred to the public SC unit.

If we assume that the patient population was distributed evenly across all 380 GPs working in PHC centres, each GP would have had 1315 appointments with 637 patients each year. Based on our series, with this patient volume in an unselected patient material, a single GP would diagnose an average of 3.2 HNCs during a 30-year career. Compared to the extremely low number of new HNC patients identified by a GP and the vast number of other patients a single GP sees each year, the median doctor-related PHC delay of 5 days seems extremely low. Most notably in this study setting, we were only able to study the doctor-related delay from the initial PHC visit until the referral to SC. Any system-related delay between the first contact with PHC and the initial visit remains unknown. The median of 1.5 visits before the referral to SC is similar to two other studies regarding HNC and oral cancer [11,12], but less than that reported in a systematic review examining oral cancer referrals [13]. Most subsequent physician visits entailed ultrasound imaging and fine-needle aspiration samples or to control symptoms. Only a single HNC patient referred from the private sector had previously visited a PHC centre for the same symptom. Thus, in general, PHC appears to effectively detect and refer potential HNC patients to SC.

In our patient population, the annual number of newly diagnosed HNC cases was 111, which is in line with the annual number reported by the Finnish Cancer Registry [14]. However, it is important to notice that oral cancer was underrepresented in the cohort consisting only patients referred from PHC centres. Most of the oral cancers were referred to SC by a dentist.

Regarding the division of labour in HNC between medical doctors and dentists, the majority of HNC patients were referred to SC by a medical doctor (72%), which agrees with a study by Ligier et al. [15]. Around one third (*n* = 40) of the HNC patients were referred from PHC. Oral cancers were underrepresented in this patient group, since the majority of these patients (64%) were referred from a public or private dentist. In a systematic review consisting of 16 studies examining the referral of oral cancer patients in PHC, fewer patients (44%) were referred by dentists [13]. This difference might stem from national differences in medical practices and the availability of different medical resources.

According to our evaluation, a new HNC was detected in 1 of every 6000 patients or 12,500 visits to PHC centres, whereby 5% of patients had HNC-related symptoms. This proportion is significantly higher than in another study by Alho et al., which investigated the incidence of HNC in Northern Finland [11]. In their study, anew HNC case was detected once in every 63,000 visits and 11% of patients who visited PHC had HNC-related symptoms. However, major differences differentiate the methods we used and those in their study. Specifically, Alho et al. randomly selected 24 different PHC centres in Finland and collected data on GP appointments for a four-week period, comparing data on HNC patients diagnosed in Northern Finland from1986 through 1996. We, on the other hand, collected data from all PHC centres in the City of Helsinki for a one-year period, and all of our HNC patients came from the same area during the same time period. These differences might explain the varying results.

According to our analysis, stratification according to certain clinical characteristics results in a higher HNC detection ratio. If patients younger than 40 years old are ruled out, the ratio rises to 1:3300. The majority of HNC patients were male (67.5%), indicating that the likelihood of a ≥40-year-old man visiting a GP in PHC would have a HNC stood at 1:2000. Luckily, patients often present with various symptoms, which point clinicians towards suspicion of a possible malignant aetiology. Among all patients who presented with a symptom possibly caused by HNC (Table 1), the ratio between HNC and non-HNC patients was 1:360. Taking into account only patients older than 40 years, the ratio increases to 1:180. Similarly, taking into account only ≥40-year-old males increases the ratio to 1:90. Among ≥40 year-old patients who reported one of the six symptoms mentioned in the MSC, the ratio of HNC to non-HNC patients was 1:107, while for ≥40-year-old males with MSC symptoms, the ratio increased to 1:63. Most notably in our study setting, we were unable to gather information on symptom duration. Thus, in this series, we were unable to evaluate the effectiveness of the 3-week rule outlined in the MSC.

In addition, certain cancer risk groups emerged. HNC was particularly prevalent among males ≥60 years old who presented with one of the six symptoms mentioned in the MSC. The likelihood of cancer among this patient group varied between 0.5 and 3%, whereby ICD-10 codes associated with a lump in the head and neck region represented the most malicious symptom (Table 1). A cancer prevalence of 3% is strikingly high considering that HNC risk factors such as tobacco smoking or heavy alcohol use were not available. In a study of over 40-year-old tobacco users (*n* = 4611), the prevalence of HNC reached nearly 3% [16]. Given these findings, it is particularly important to consider HNC as a possible cause for these symptoms if the symptoms are unexplained by an acute respiratory tract infection or other distinct aetiology. This is particularly important if the patient has a history of tobacco use and/or heavy alcohol consumption and symptoms persisted for at least three weeks. Moreover, this also suggests that the MSC guidelines for patients and GPs are well-founded. Furthermore, the ratio might be even higher if we had ICD codes specific to a neck mass. Our data do not include the number of patients with a probable HNC-related symptom any individual SC specialist encountered; thus, the ratio in a selected series at specialist outpatient clinics is likely higher. A British study on a 2-week wait fasttrack referral system implemented to reduce delays in patients with suspected HNC found that 9.5% of patients referred through the system had HNC [17].

One limitation of this study lies in the likely heterogeneity in the use of ICD-10 codes. Because determining the ICD-10 diagnosis code relies on a subjective decision-making process from the treating physician, the comparability of diagnoses remains limited. In 17.5% of our cases, the ICD-10 diagnosis code was not used at all or was non-descriptive. This generates a bias when assessing the relative cancer risks among different patient groups. Importantly, the number of doctor visits with a certain symptom-related ICD-10 diagnosis code does not reflect the prevalence of specific symptoms in the general population. That is, a distinctive difference exists between a patient with a symptom and a patient seeking medical advice because of a symptom, which should alarm GPs, particularly if the symptom is potentially cancer related and the patient has risk factors for a malignancy. Furthermore, it is important to notice that usually only a single ICD-10 code is used per visit; if patient visits PHC with multiple symptoms, only the primary symptom is recorded. Therefore, calculations which are too precise or far-fetched conclusions should not be made. Another apparent limitation is that the codes include a large variety of different and often benign conditions, particularly in the most commonly used codes, such as difficulties breathing, pain in the throat or painful swallowing and a blocked nose. These ICD-10 codes can be used to describe anything from cardiac arrest to a viral upper respiratory tract infection. In addition, further limitations include the lack of data on tobacco use and alcohol consumption. Despite these obvious limitations related to the use of ICD-10 codes, they are currently the only practical way to study symptom epidemiology in a large population series before the emergence of structured patient record systems.

To conclude, we identified one HNC patient for every 6000 patients visiting PHC. If patients’ symptoms, age and sex were taken into account, this ratio climbed to as high as 1:33 (≥60-year-old male with a lump in the head and neck region). Despite the challenges in identifying HNC patients from the normal patient flow, the median PHC delay was only 5 days and delays of over a month occurred only in 12.5% of all cases.

## Ethical approval

All procedures performed in studies involving human participants were in accordance with the ethical standards of the institutional and/or national research committee (The Research Ethics Board at the Hospital District of Helsinki and Uusimaa, record number: 398/13/03/02/15) and with the 1964 Helsinki declaration and its later amendments or comparable ethical standards.

## References

[1] Koivunen P, Rantala N, Hyrynkangas K, et al. The impact of patient and professional diagnostic delays on survival in pharyngeal cancer. Cancer. 2001;92(11):2885–2891. 11753962 10.1002/1097-0142(20011201)92:11<2885::aid-cncr10119>3.0.co;2-g

[2] Murphy CT, Galloway TJ, Handorf EA, et al. Survival impact of increasing time to treatment initiation for patients with head and neck cancer in the united states. J Cin Oncol. 2016;34(2):169–178. 10.1200/JCO.2015.61.5906 PMC4858932 26628469

[3] Teppo H, Alho OP. Relative importance of diagnostic delays in different head and neck cancers. Clin Otolaryngol. 2008;33(4):325–330. 18983341 10.1111/j.1749-4486.2008.01704.x

[4] van Harten MC, Hoebers FJ, Kross KW, et al. Determinants of treatment waiting times for head and neck cancer in the Netherlands and their relation to survival. Oral Oncol. 2015;51(3):272–278. 25541458 10.1016/j.oraloncology.2014.12.003

[5] Waaijer A, Terhaard CH, Dehnad H, et al. Waiting times for radiotherapy: consequences of volume increase for the TCP in oropharyngeal carcinoma. Radiother Oncol. 2003;66(3):271–276. 12742266 10.1016/s0167-8140(03)00036-7

[6] World Health Organization. Globocan 2018, estimated cancer incidence, mortality and prevalence worldwide in 2018; 2018 [cited 2020 Sep 2]. Available from: http://gco.iarc.fr/today/online-analysis-table?v=2018&mode=cancer&mode_population=continents&population=900&populations=900&key=asr&sex=0&cancer=39&type=0&statistic=5&prevalence=0&population_group=0&ages_group%5B%5D=0&ages_group%5B%5D=17&nb_items=5&group_cancer=1&include_nmsc=1&include_nmsc_other=1.

[7] Lyberg-Ahlander V, Rydell R, Fredlund P, et al. Prevalence of voice disorders in the general population, based on the Stockholm public health cohort. J Voice. 2019;33(6):900–905. 30126692 10.1016/j.jvoice.2018.07.007

[8] Palmer JN, Messina JC, Biletch R, et al. A cross-sectional, population-based survey of U.S. adults with symptoms of chronic rhinosinusitis. Allergy Asthma Proc. 2019;40(1):48–56. 30582496 10.2500/aap.2019.40.4182

[9] European Head & Neck Society. Make sense campaign – head & neck cancer; 2020 [cited 2020 Sep 2]. Available from: https://makesensecampaign.eu/en/cancer-information/head-neck-cancer.

[10] Nieminen M, Aro K, Jouhi L, et al. Causes for delay before specialist consultation in head and neck cancer. Acta Oncol. 2018;57(12):1677–1686. 30141700 10.1080/0284186X.2018.1497297

[11] Alho OP, Teppo H, Mantyselka P, et al. Head and neck cancer in primary care: presenting symptoms and the effect of delayed diagnosis of cancer cases. CMAJ. 2006;174(6):779–784. 16534084 10.1503/cmaj.050623 PMC1402394

[12] Crossman T, Warburton F, Richards MA, et al. Role of general practice in the diagnosis of oral cancer. Br J Oral Maxillofac Surg. 2016;54(2):208–212. 26682494 10.1016/j.bjoms.2015.11.003

[13] Grafton-Clarke C, Chen KW, Wilcock J. Diagnosis and referral delays in primary care for oral squamous cell cancer: a systematic review. Br J Gen Pract. 2019;69(679):e112–e126. 30455220 10.3399/bjgp18X700205 PMC6355296

[14] Malila N, Pitkäniemi J, Virtanen A. Finnish cancer registry, cancer; 2016 [cited 2020 Sep 2]. Available from: https://syoparekisteri.fi/assets/files/2019/02/vuosiraportti_2016.pdf.

[15] Ligier K, Dejardin O, Launay L, et al. Health professionals and the early detection of head and neck cancers: a population-based study in a high incidence area. BMC Cancer. 2016;16:456. 27406036 10.1186/s12885-016-2531-7 PMC4942882

[16] Prout MN, Sidari JN, Witzburg RA, et al. Head and neck cancer screening among 4611 tobacco users older than forty years. Otolaryngol Head Neck Surg. 1997;116(2):201–208. 9051065 10.1016/S0194-59989770326-7

[17] Hong B, Shaikh Z, Adcock S, et al. Two-week wait false alarms? A prospective investigation of 2WW head and neck cancer referrals. Br Dent J. 2016;220(10):521–526. 27228932 10.1038/sj.bdj.2016.376

